# Pure nongestational choriocarcinoma of the ovary: a case report

**DOI:** 10.1186/1477-7819-11-7

**Published:** 2013-01-15

**Authors:** Youn Jin Choi, Keun Young Chun, Yong Wook Kim, Duck Yeong Ro

**Affiliations:** 1 Department of Obstetrics and Gynecology, College of Medicine, the Catholic University of Korea, Incheon St. Mary’s Hospital, Incheon Si, Bupyeonggu 665 Bupyeongdong, Incheon, 403-702, South Korea

**Keywords:** Chemotherapy, Nongestational choriocarcinoma, Ovary

## Abstract

Pure ovarian choriocarcinoma can be gestational or nongestational in origin. Nongestational choriocarcinoma of the ovary is extremely rare, and its diagnosis is very difficult during the reproductive years. We present a case of a 33-year-old woman diagnosed with pure nongestational ovarian choriocarcinoma. Following surgery, multiple courses of a chemotherapy regimen of etoposide, methotrexate, and actinomycin-D (EMA) were effective.

## Background

Choriocarcinoma of the ovary is a rare aggressive tumor, which may be either gestational or nongestational in origin. Most occurrences are gestational in origin and usually metastasize to the ovary from a uterine or tubal choriocarcinoma. In contrast, pure nongestational choriocarcinoma of the ovary (NGCO) is extremely rare, and diagnosis is very difficult in women of reproductive age [1,2]. We present a case of pure NGCO occurring in a 33-year-old woman with a brief review of the literature.

## Case presentation

A 33-year-old woman (gravida 4, para 2) presented to our hospital with complaints of lower abdominal pain and vaginal bleeding for several days. Her last menstrual period occurred three weeks before her presentation, and previous menstrual cycles were regular. Her husband had undergone vasectomy several years ago. Her medical history and family history were unremarkable. The general condition of the patient appeared to be good; however, pelvic examination revealed a mass in the left ovary. Chest radiograph was normal. The urine test result for human chorionic gonadotropin (hCG) was positive, and serum β-hCG level was 74,612 mIU/ml. Transvaginal ultrasound (TVS) revealed a left adnexal mass of 5 × 4 cm with profuse abdominal fluid accumulation.

**Figure 1:**
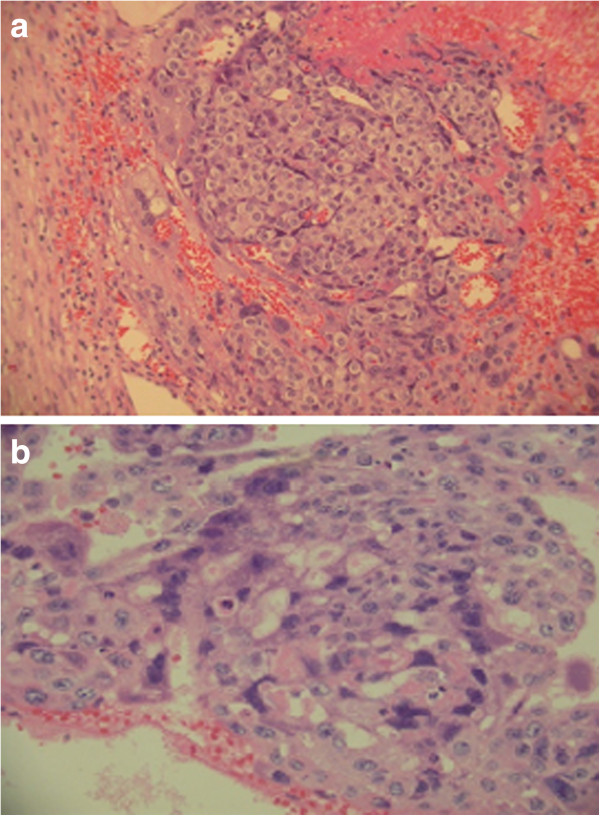
**Histological stain of the left ovary with plexiform pattern of triphasic differentiation into cytotrophoblast, syncytiotrophoblast, and intermediate trophoblast and marked cytologic atypia and hemorrhage with intracytoplasmic hyaline material.** Hematoxylin and eosin (H&E) stain. **(a)** 200×. **(b)** 400×.

According to a diagnosis of left tubal pregnancy, laparoscopic exploration was performed. The left ovary was as large as a tennis ball and was ruptured and surrounded by a hematoma. A cystic mass of 2 × 2 cm was found on the right ovary and both fallopian tubes were intact. A 3-cm white-yellow mass and a 1-cm nodule were scattered in the peritoneal cavity. Approximately 500 ml of intraperitoneal blood was noted. Left oophorectomy, right ovarian cystectomy, and peritoneal mass biopsies were performed. On the second and fifth postoperative day, serum β-hCG levels were 17,963 mIU/ml and 10,172 mIU/ml, respectively. Histological examination showed a choriocarcinoma of the left ovary and corpus luteal cyst of the right ovary. Peritoneal biopsies showed ovarian tissue with marked central change of the corpus albicans and partial covering of choriocarcinomatous nests. The tumor cells were composed of cytotrophoblastic cells forming clusters of cytotrophoblast separated by streaming masses of syncytiotrophoblasts (Figure 1). The tumor mass was diffusely hemorrhagic and necrotic. Tumor cells were found to infiltrate the stroma, but the external capsule was free from malignant invasion. One week after surgery, the levels of tumor markers, including alpha-fetoprotein and cancer antigen 125, were within normal limits. Chest computed tomography and pelvic magnetic resonance imaging showed no evidence of lung metastasis or lesion remnants.

Endometrial biopsy was performed to rule out gestational origin. Histological examination of the endometrium revealed few fragments of basal endometrium with focal, early proliferative-phase regions without trophoblastic lesions. The patient received nine courses of EMA chemotherapy, including etoposide (100 mg/m^2^), methotrexate (100 mg/m^2^), and actinomycin-D (0.5 mg), at seven-day intervals without major complications. After six courses of chemotherapy, the β-hCG level decreased to normal (2.74 mIU/ml) and remained normal thereafter. A further three courses were completed for consolidation. The patient remains without evidence of disease five years after initial diagnosis.

Choriocarcinoma arises in the ovary and may be of either gestational or nongestational origin. Gestational choriocarcinoma of the ovary (GCO) can occur following pregnancy and is characterized by presentation in the uterine cavity [3]. NGCO most commonly arises from ovarian germ cell tumors but can originate from any epithelial cancer, including cancer of the lung, stomach, and bowel [4]. It is important to distinguish between these types because NGCO is generally believed to have a poor prognosis. However, no distinctive ultrastructural or immunohistochemical differences have been reported between choriocarcinomas of gestational and nongestational origin [1]. Clinical diagnosis of NGCO is often difficult, not solely owing to its rarity. Pure NGCO is pathologically indistinguishable from GCO, except in patients who are unable to conceive or who have never had sexual intercourse [5]. Moreover, the symptoms are often nonspecific and can mimic other more common conditions that occur in young women, such as a hemorrhagic ovarian cyst, tuboovarian abscess, ovarian cyst torsion, and ectopic pregnancy [6]. The symptoms of vaginal bleeding, elevated hCG level, pelvic pain, and an adnexal mass often lead to incorrect diagnosis of ectopic pregnancy, threatenedor incomplete abortion, cervical polyp, or other types of malignancy [7]. In this case, the ultrasonographic findings, clinical symptoms and signs, and rarity of choriocarcinoma mistakenly led to the initial diagnosis of ectopic pregnancy.

Saito *et al*. first described the diagnostic criteria for NGCO in 1963. These include absence of disease in the uterine cavity, pathological confirmation of disease, and exclusion of molar pregnancy and of intrauterine pregnancy [8]. All the criteria were fulfilled in this case. Owing to the rarity of the condition, little information on clinicopathological features and therapeutic options is available. GCO has been treated by methotrexate-based chemotherapy, but some studies showed that NGCO may be resistant to this chemotherapy, requiring more aggressive combination chemotherapy [9]. We began adjuvant chemotherapy with the EMA (etoposide, methotrexate, actinomycin-D) regimen. After six courses of chemotherapy, the serum β-hCG level was normalized.

A comparison of case reports of ovarian choriocarcinoma is presented in Table 1. The summarized cases show limitations in that they are not all fully staged and the chemotherapy protocols are diverse. Analysis of the seven cases documented thus far suggests that the disease responds well to the combination of surgery and postoperative adjuvant chemotherapy. However, long-term effects of such therapy should be further studied with more cases.

**Table 1:** Nongestational choriocarcinoma of the ovary: a summary of cases

**Authors (Reference)**	**Age**	**β-human chorionic gonadotropin (mIU/ml)**	**Surgery**	**Chemotherapy**	**Outcome**
Gerson [10]	33	564,000	Right salpingo-oophorectomy	Etoposide, methotrexate, actinomycin-D, cyclophosphamide, vincristine	No evidence of disease
Yamamoto [11]	19	206,949	Left oophorectomy	Etoposide, methotrexate, actinomycin-D	No evidence of disease
Balat [9]	24	8,968	TAH, BSO, PLND, partial omentectomy, and sternum mass excision	Bleomycin, etoposide, cisplatin	Died during chemotherapy
Roghaei [12]	47	970	TAH, BSO, PLND, and partial omentectomy	Etoposide, methotrexate, actinomycin-D, cyclophosphamide, vincristine	No evidence of disease
Byeun [13]	28	13,378	Right salpingo-oophorectomy	Etoposide, methotrexate, actinomycin-D	No evidence of disease
Corakci [14]	22	15,050	TAH, BSO, PLND, and PaLND	Bleomycin, etoposide, cisplatin	No evidence of disease
Lyn [15]	48	7,663	TAH, BSO, PLND, PaLND, omentectomy, appendectomy, and peritoneal biopsy	Bleomycin, etoposide, cisplatin	No evidence of disease

BSO, bilateral salpingo-oophorectomy; PaLND, paraaortic lymph node dissection; PLND, pelvic lymph node dissection; TAH, total abdominal hysterectomy.

## Conclusions

Because of the small number of patients with pure ovarian choriocarcinoma, a consensus on the treatment regimen including surgery and chemotherapy is lacking. Our report adds data that can be helpful in both diagnosis and treatment of this rare disease.

## Consent

Written informed consent was obtained from the patient for publication of this report and accompanying images. A copy of this written consent is available for review by the Editor-in Chief of this journal.

## Abbreviations

EMA: etoposide, methotrexate, and actinomycin-D; GCO: gestational choriocarcinoma of the ovary; hCG: human chorionic gonadotropin; NGCO: nongestational choriocarcinoma of the ovary; TVS: transvaginal ultrasound.

## Competing interests

The authors declare that they have no competing interests.

## Authors’ contributions

YJC made substantial contributions to conception and design, and acquisition of data. She also drafted the manuscript and revised its final form. KYC and YWK were involved in analysis and interpretation of data. DYR contributed to interpreting the data and gave final approval of the version to be published. All authors read and approved the final manuscript.
